# COVID-19 and Pediatric Lung Disease: A South African Tertiary Center Experience

**DOI:** 10.3389/fped.2020.614076

**Published:** 2021-01-20

**Authors:** Diane M. Gray, Mary-Ann Davies, Leah Githinji, Michael Levin, Muntanga Mapani, Zandiswa Nowalaza, Norbertta Washaya, Aamir Yassin, Marco Zampoli, Heather J. Zar, Aneesa Vanker

**Affiliations:** ^1^Department of Pediatrics and Child Health, University of Cape Town, Cape Town, South Africa; ^2^Medical Research Council (MRC) Unit on Child and Adolescent Health, University of Cape Town, Cape Town, South Africa; ^3^School of Public Health and Family Medicine, University of Cape Town, Cape Town, South Africa

**Keywords:** COVID-19, SARS-CoV-2, pediatrics, lung disease, chronic lung disease in childhood, low middle-income countries, pediatric tuberculosis

## Abstract

The COVID-19 pandemic led to rapid global spread with far-reaching impacts on health-care systems. Whilst pediatric data consistently shown a milder disease course, chronic lung disease has been identified as a risk factor for hospitalization and severe disease. In Africa, comprised predominantly of low middle-income countries (LMIC), the additional burden of HIV, tuberculosis, malnutrition and overcrowding is high and further impacts health risk. This paper reviewed the literature on COVID-19 and chronic lung disease in children and provides our experience from an African pediatric pulmonary center in Cape Town, South Africa. South African epidemiological data confirms a low burden of severe disease with children <18 years comprising 8% of all diagnosed cases and 3% of all COVID-19 admissions. A decrease in hospital admission for other viral lower respiratory tract infections was found. While the pulmonology service manages children with a wide range of chronic respiratory conditions including bronchiectasis, cystic fibrosis, asthma, interstitial lung disease and children with tracheostomies, no significant increase in COVID-19 admissions were noted and in those who developed COVID-19, the disease course was not severe. Current evidence suggests that pre-existing respiratory disease in children does not appear to be a significant risk factor for severe COVID-19. Longitudinal data are still needed to assess risk in children with immunosuppression and interstitial lung diseases. The indirect impacts of the pandemic response on child respiratory health are notable and still likely to be fully realized and quantified. Ensuring children have access to full preventive and care services during this time is priority.

## Background

The COVID-19 pandemic caused by the novel coronavirus (SARS-CoV-2), commenced in Wuhan, China in late 2019 with rapid global spread, sparing no continent, with the first African case being reported in Egypt in February 2020. By 29 September 2020, 33,249,563 cases globally had been reported with 1,000,040 deaths, with 216 countries affected. Africa had reported 1,179,152 cases and 25,760 deaths, 3.5 and 2.6% of the global burden, respectively (1). As the pandemic spread, with high mortality, predominantly from respiratory disease, and significant stress on healthcare systems, a number of concerns were anticipated for the World Health Organization (WHO) Africa region where HIV prevalence and other infectious diseases such as tuberculosis (TB) remains high and many countries still depend on fragile healthcare systems (2). In Africa, South Africa has reported the highest number of confirmed corona virus cases to date (September 2020) (1). The pediatric data from around the world has consistently showed a lower infection rate and milder disease course in children. In a review of over 70 000 Chinese cases of COVID-19, <1% were children under 10 years of age with different and less severe symptoms to that in adults (3). Similarly, a very large analysis of nearly 150 000 COVID-19 cases in the United States of America found only 1.7% were children <18 years of age, with a very small number requiring ICU admission and extremely low mortality (4). However, multi-center data from over 400 children in Latin-America showed a more severe form of COVID-19, with 12.7% of children included requiring ICU admission and a high proportion of multi-system inflammatory syndrome (MIS-C) diagnosed, concluding more severe disease form in Latin/Hispanic children or those of lower socioeconomic level (5). Other low and middle income countries (LMIC) have also reported fewer and less severe COVID-19 cases in children compared to adults (6). Chronic lung disease has been one of the commonest pre-existing medical conditions reported in hospitalized pediatric COVID-19 cases, both in Europe and in South Africa (7, 8). It has been identified as a risk factor for severe COVID-19 disease and death in adults (9, 10). In children and adolescents chronic respiratory illness has been associated with admission to an intensive care unit (ICU), chronic respiratory illness conferring an increased risk of OR 3.1 (CI 1.2–8.2) for pediatric ICU admission (7). Similarly in South Africa, underlying asthma and chronic respiratory illness was a commonly reported underlying risk factor in children admitted with COVID-19 (8). This has caused increased anxiety for parents and children with chronic lung disease (11). Highlighting the importance of better understanding risk for these children. The impact of pre-existing lung disease on COVID-19 disease severity has been a particular concern in LMIC where there is a high burden of additional risk factors for severe respiratory disease such as HIV, TB, malnutrition and overcrowding (12, 13).

In addition, the indirect effects of COVID-19 on child health are not inconsequential (6, 14). Measures to curb the spreads of SARS-CoV2 infection has led to many countries, including South Africa, implementing “lock down” policies with far-reaching socio-economic consequences including restrictions to routine healthcare access (2, 15–17). Diversion of healthcare resources to focus on COVID-19 care, including reducing routine chronic clinic services and primary healthcare facility access has meant that children have had limited access to routine care and even immunization programmes (16–18). Fear of contracting COVID-19 from healthcare facilities has further hampered healthcare attendance. The impact of reduced essential maternal and child health interventions from COVID-19 in LMIC is estimated to contribute an increase of 9.8–44.7% in under-5 child deaths per month, and an 8.3–38.6% increase in maternal deaths per month (14).

In this paper we aim to review the literature on COVID-19 in children with chronic lung disease globally and give our experience from an African pediatric pulmonary center in Cape Town, Western Cape Province, South Africa.

## Epidemiology of COVID-19 in South Africa

Although there is no public global database with age disaggregated data and only 42 countries (about 1 in 4) with age disaggregated COVID-19 publicly available data, the burden of COVID-19 cases and severe disease in children <20 years age is widely recognized to be less than that of adults, but varies considerably across countries from 1% of all cases in Spain, to 23% in Paraguay (19). There is extremely limited more granular age-disaggregated country-level COVID-19 data. Surveillance studies using antibody testing of all SARS-CoV-2 infections by age group have been relatively limited to date with some suggesting lower infection rates in children, while others found no difference (20–22). In South Africa, by 5 September 2020, children <20 years accounted for ~8% of 638,517 confirmed COVID-19 positive cases, with 15–19 year olds accounting for nearly half of these. The cumulative incidence risk of laboratory confirmed COVID-19 cases per 100,000 increased with age in children from 130 (age <5 years) to 520 (15–19 years), and was considerably lower than the cumulative incidence for the country as a whole of 1,086 cases/100,000 (8).

Data from the Western Cape province of South Africa confirms a very low burden of severe COVID-19 disease in children. As of 25 September 2020, there were 6,336 COVID-19 positive cases in children aged <20 years comprising 6% of all diagnosed cases in the province, 658 hospital admissions, 27 ICU admissions and 24 deaths (0.6% of all COVID-19 deaths in the province) giving a diagnosed case fatality ratio of 0.4%. The number of cases over time followed that of adults with a peak of newly diagnosed cases in late June 2020 and subsequent gradual decline. Multisystem inflammatory syndrome in children (MIS-C) has recently become a notifiable disease in South Africa. A case series of 23 MIS-C cases from the 2 tertiary hospitals providing pediatric care in Cape Town has been published with no deaths (23).

Several modeling studies have suggested that although COVID-19 itself is mostly a mild disease in children, the indirect effect of COVID-19 due to service disruptions may have substantial impacts on maternal and child health with possible increases in the incidence of several diseases and of child death. National immunization coverage dropped during South Africa's lockdown from 82% in April 2019 to 61% in April 2020 with particularly concerning declines in measles coverage from 77 to 55%, especially in the Western Cape where COVID-19 cases initially increased most rapidly and measles vaccine coverage in April 2020 was only 48% (18). Concerns have also been raised about reduced coverage of contraception, reduced safe pregnancy terminations, reduced HIV diagnosis and ART coverage in pregnancy with possible increased mother to child transmission, and increased malnutrition due to the severe economic consequence of lockdown (15).

## Planning and Preparation for Pandemic and Implications on Respiratory Care

The Red Cross War Memorial Children's Hospital (RCWMCH) in Cape Town is a dedicated tertiary and referral hospital that offers a wide range of specialist pediatric services and is affiliated to the University of Cape Town, South Africa (24). The pediatric pulmonology unit manages children with a wide range of both acute and chronic respiratory conditions thorough in-patient and outpatient services. The COVID-9 pandemic has required an adaptation of services to allow for continued care of children with respiratory condition during this time (25).

In South Africa an early “hard” lockdown was accompanied by an early and widespread adoption of public health measures including social distancing, strict hand-sanitizing, and mask wearing. Routine public transport was halted, non-emergency health services were limited to shift resources to COVID-19 screening and treatment centers and social service offices were closed; a response that has been credited in part for lower case numbers and mortality rates compared to Europe and USA. In the pediatric hospital this included cancellation of non-essential visits, limited radiology services, cessation of lung function testing, closing of pediatric beds, shifting of staff to adult and COVID-19 services and in-hospital cohorting pathways that reduced risk of COVID-19 infection spread, at the expense of continuity of care. All factors that could impact on timely diagnosis, management and follow-up of respiratory disease.

## COVID-19: Impact on Acute Lower Respiratory Tract Infections in Children

A notable drop in pediatric admissions for acute lower respiratory tract infections (LRTI) other than COVID-19, particularly viral LRTI, has been described in a number of countries (26–28). In the Southern Hemisphere, the COVID-19 pandemic coincided with the annual 2020 viral respiratory season. Pediatric ICU (PICU) admission data from a large Latin-American registry showed 83% fewer PICU admissions for LRTIs in 2020 compared to the 2018/2019 average over the same period, especially for respiratory syncytial virus and influenza (29). South Africa has similarly seen markedly decreased incidence and hospitalization rates for LRTI through the usual winter admission surge (Olsen et al., under review). RSV-LRTI in particular has been markedly reduced compared to the preceding 5 years. A similar vastly reduced incidence of influenza and influenza related hospitalization has been seen, with no pediatric hospital admissions for influenza since May 2020. This marked reduction in expected LRTI cases has also been noted in China and Hong Kong, with a precipitous drop in viral-associated infections coinciding with the onset of the initiation of COVID-19 related public health measures (26).

## COVID-19 in Children with Pre-Existing Lung Disease and Co-Morbidities (Table 1)

### Bronchiectasis and Bronchiolitis Obliterans

Individuals with pre-existing chronic lung disease, including chronic obstructive pulmonary disease (COPD), are at increased risk for severe COVID-19, in adults (10, 41). Bronchiolitis obliterans (BO) is a chronic obstructive airway disease characterized by airflow obstruction of the small airway. Injury to airway epithelium leads to neutrophilic inflammatory changes and scarring and eventual progression to chronic obstructive lung disease (42). Patients with BO are at risk of severe exacerbations with intercurrent respiratory infections, hence the concern for increased risk of more severe COVID-19 (43). In addition, patients with chronic lung diseases have increased epithelial expression of ACE2 receptors where SARS-CoV-2 binds to gain access into the cells (44); and increased morbidity and mortality in chronic lung disease may be compounded by pre-existing reduced lung reserve (10). Pediatric cohort studies have described chronic airway obstruction as a comorbidity associated with COVID-19 severity (30, 31). Underlying chronic obstructive airway disease, most commonly asthma and bronchopulmonary dysplasia, has been reported in 5% of children <18 years in Europe admitted with COVID-19 and 13% of children requiring ICU admission (7). Bronchiectasis was uncommon which reflects the low prevalence if these conditions in HIC.

**Table 1:** COVID-19 in children with chronic respiratory illness and respiratory co-morbidities.

**Chronic lung disease**	
Bronchiolitis obliterans	Chronic obstructive lung disease associated with severe COVID-19 in European children (7, 30). Chronic lung disease a common co-morbidity in those admitted with COVID-19 in Europe (7, 31).
Bronchiectasis	No published data on bronchiectasis in pediatric COVID-19.
Asthma	No increased risk for COVID-19 related hospitalization nor ICU admission (32, 33). Decreased asthma admissions reported in some centers (34, 35). Uncontrolled asthma a risk for COVID-19 related mortality adults (10); highlighting importance of good asthma control.
Cystic fibrosis	Not associated with severe COVID-19 disease (36, 37).
Interstitial lung disease	Impact in children with ILD unknown. Increased COVID related mortality in adults with ILD suggests caution (38). Severe COVID-19 pneumonia is a cause of diffuse alveolar damage and fibrosis. Restrictive lung function and decreased gas diffusion reported in adults after severe COVID-19 pneumonia (39).
**Co-morbidities**	
Tracheostomies	No published data on increased risk for COVID-19 infection or severe disease in children with tracheostomies. Any risk likely associated with underlying condition
Tuberculosis	Tuberculosis, current or previous, has been associated with COVID-19 morbidity in South African adults (40); data not currently available in children. Clinical experience in children to date does not indicate that tuberculosis, infection or disease, is a major risk factors for COVID-19 morbidity and mortality. Data analysis is ongoing.
HIV infection	HIV infection is risk factor for COVID-19 severity in Africa (40); data not currently available in children. Clinical experience in children to date does not indicate that HIV nor HIV exposure are major risk factors for COVID-19 morbidity and mortality. Data analysis is ongoing.

The respiratory out-patient clinic at RCWMCH, follows up a cohort of approximately 120 children with bronchiectasis and/or BO. Since the first case of COVID-19 was reported and lockdown measures initiated in South Africa in March 2020, patients were preferentially followed up telephonically. During this time, the pulmonology division did not experience any up-surge in bronchiectasis patient admission nor increased exacerbations, with none of these patients requiring admission with COVID-19. Data on pediatric COVID-19 admissions from multiple South African centers is currently being collected and will assist in better understanding the prevalence and risk.

### Asthma

Asthma is a common chronic disease in children in many African countries. The prevalence of childhood asthma in Africa is higher than the global average and the burden of childhood asthma in Africa is increasing (45). At the beginning of the pandemic asthma was considered a risk factor for severe COVID-19. However, emerging data has not found asthma to increase risk of hospitalization nor ICU admission with COVID-19 infection (32, 33). In a recent European Respiratory Society survey of 94 centers including 945 children with COVID-19, 49 (5%) children were asthmatic, most children had very mild symptoms and 20% presented with classic asthma exacerbations, potentially triggered by coronavirus. Although 9% of asthmatic children required ICU admission, all recovered with average length of hospital stay 6.9 ± 5.2 days (46). Some centers have in fact reported decreased asthma admissions during this period; probably a combination of less exposure to infectious triggers during lockdown, improved adherence due to fear of getting ill and avoidance of attending health services (34, 35). We experienced a similar decrease in asthma admissions during the early strict lockdown, and have not seen the expected delayed spike in poor control. However, despite SARS-CoV-2 appearing not to commonly cause wheezing illness nor severe illness (47), there is a theoretical possibility that asthmatics may have attacks triggered by COVID-19 infection; good asthma control is essential as a general principle and as a precautionary measure during this time.

### Cystic Fibrosis

People with cystic fibrosis (CF) were considered at risk of severe COVID-19 especially those with advanced lung disease based on past experience of CF and the *influenza* A (H1N1) pandemic in 2010 (48). However, an early report from 8 countries of 40 cases of COVID-19 in people with CF has dispelled this initial concern (36). The majority were adults (youngest age 15 years), 38% had CF-related diabetes and 11 were lung transplant recipients. Thirteen (32%) patients required oxygen therapy and one (a lung transplant recipient) needed mechanical ventilation. There were no deaths reported in this series. European CF Society registry data reported (as of 10 September 2020), 144 COVID-19 cases in people with CF, of which only 36 were <20 years and all had mild illness or were asymptomatic (37). South Africa has so far documented only 3 cases of COVID-19 in people with CF (49). One of the speculated reasons for the low incidence of COVID-19 in the CF population is their pre-existing behavior of taking precautions to avoid infections. Preventing infection is an important principle in CF care which preceded the pandemic. However, a greater concern is the detrimental impact on CF health caused by disruption to routine CF care. Cystic fibrosis is a complex disorder that requires multidisciplinary care and close monitoring. Typically, patients are seen every 1–3 months for routine check-ups, lung function and sputum tests. In addition, treatment consists of daily chest physiotherapy exercises and nebulized medications e.g., antibiotics. Since COVID-19 lockdown restrictions were implemented, routine medical care has been severely disrupted, including routine referrals for diagnostic tests and suspension of lung transplant programs. Furthermore, most patients and families are avoiding health facilities where possible. Certain procedures common in CF patients such as lung function tests, collecting sputum samples and chest physiotherapy with nebulizers are considered high risk due to increased aerosolization of respiratory droplets. While it is reasonable to defer routine checkups in some, the health and well-being of the more vulnerable or ill patient may be compromised. This concern extends to CF research worldwide where many active clinical trials have been suspended (50). Guidelines and recommendations for CF care in South Africa during COVID-19 pandemic have been published covering all aspects from how to prevent SARS-CoV-2 infections, through to reducing the risk of spreading SARS-CoV-2 and returning to school or the workplace (51); highlighting re-instatement of routine CF care with infection control measures as priority.

### Interstitial Lung Disease

There is scarcity of data on the impact of COVID-19 ion children with interstitial lung disease (ILD), especially in LMICs. A European multicenter audit of patients older than 19 years with a diagnosis of pre-existing ILD, hospitalized due to COVID-19, showed a high mortality of 39.3% from COVID-19 in patients with underlying ILD, compared to 15.4% in age, sex, disease severity matched COVID-19 patients without pre-existing ILD (38). The severity of disease in these patients is most likely related to a combination of COVID-19 causing exacerbating ILD, exaggerated inflammatory responses and coagulation abnormalities (38). The use of immune-suppressive agents, which are the mainstay of therapy in ILD, have not been consistently associated with severe COVID-19 disease (52). In fact use of dexamethasone has been found to improve survival and used in the management of COVID-19 pneumonia and acute respiratory distress syndrome (53).

Severe COVID-19 pneumonia can itself result in interstitial fibrosis. COVID-19 pneumonia has a histopathological picture described as diffuse alveolar damage of acute respiratory distress syndrome, with fibrosis as an end complication. Impaired gas diffusion and restrictive lung function are the commonest lung function abnormalities reported in adult cohort studies post COVID-19 pneumonia. Lung function impairment being related to disease severity (39). This is an important consideration in the long-term follow-up of survivors of severe COVID-19 disease (54).

Similar to the global data, in our institution, none of our patients with ILD were admitted with COVID-19. However, data on the impact of COVID-19 on children with pre-existing ILD is needed. Given the adult data, it is reasonable to consider these children at increased risk and to advise strict infection control measures.

### Tracheostomies

Tracheostomy insertion and routine tracheostomy care are aerosol generating procedures and therefore high risk for COVID-19 transmission, particularly to health care workers (55). Most of the literature on tracheostomy insertion and care during the COVID-19 pandemic is in adults and from high income settings. Guidelines have been published on safe tracheostomy procedure and care in a COVID-19 patient (55–57). This is of importance as many COVID-19 patients require prolonged ventilation and benefit from tracheostomy insertion (55, 56). Additional measures that can be utilized to limit virus transmission in tracheostomy routine care include the use of cuffed tracheostomy tubes, closed suctioning circuits and heat and moisture filters (55, 57).

The RCWMCH's Breatheasy program, is a holistic multidisciplinary programme that manages children who require home-based tracheostomy care and/or long-term ventilation (58, 59). As of September 2020, the programme was managing a total of 202 children. Due to the COVID-19 pandemic ambulatory clinics in this service moved to a telehealth platform with only essential hospital visits being made. Only two patients tested positive for COVID-19 and both had mild symptoms; both had a close family member as the contact. This suggests that despite children with tracheostomies in general being at increased risk of lower respiratory tract infections (60), severe COVID-19 was not observed in our patient cohort.

One of the strengths of the Breatheasy© program is that it facilitates weekly support groups for caregivers of children admitted to hospital (59). This creates a strong psychosocial support structure, particularly for caregivers of children with new tracheostomies interacting with more experienced caregivers. To facilitate social distancing this support group was suspended with negative effects evidenced by increased parental emotional and psychological stress. Caregiver anxiety was further compounded by hospital regulations, that limited movement of caregivers in and out of hospital as well as hospital visitors. These limitations have had significant impact on family dynamics, especially in families whose children required prolonged hospitalization for training in tracheostomy care.

## Tuberculosis (TB) and Human Immunodeficiency Virus (HIV)

Sub-Saharan Africa is facing the pre-existing dual-epidemic of TB and HIV, in addition to the COVID-19 pandemic. Globally, 10 million people developed TB disease in 2018, of these 1.1 million were children, the majority from sub-Saharan Africa (61). Of the 1.8 million children living with HIV, 90% are in sub-Saharan Africa (62). HIV infected children are at an increased risk for acute and chronic respiratory conditions and may be more susceptible to severe COVID-19 disease. In addition HIV exposed uninfected infants are at risk of more severe lower respiratory tract infections with worse outcomes in early life (63) and similairly may be more suscpetible to COVID-19. TB disease is associated with chronic respiratory illness, raising concerns that it too will be associated with increased risk of COVID-19 and of severe disease. Conversely, having had TB may in fact upregulate innate immunity boosting immune responses to COVID-19, the theory for why BCG vaccine may be partially protective for severe disease (64). BCG given at birth does not however appear to confer protection against SARS-COV-2 infection (65), although multicentre studies are ongoing. It is unclear if latent tuberculosis infection and co-infection with SARS-COV-2 induces immune storm but two case studies in China reported use of immune modulators in adult patients with LTBI co-infected with COVID-19 was useful (66).

Initial data from Africa suggests that HIV is a risk factor for COVID-19 severity in adults infected with HIV, although not a consistent finding globally (40). A case series from Italy did not report HIV as a predictor for severe COVID-19 nor death especially in patients who were virally suppressed (67). A recent meta-analysis showed increased odds for severe COVID-19 disease in patients with immunosuppression (776 patients; OR = 3.29, 95% CI: 0.89–12.21, *P* = 0.075), though the causes of immunosuppression reported in this paper were multifactorial not just HIV (68). In Western Cape, South Africa, HIV and current or previous TB were both independently associated with COVID-19 related death in adults, with HIV adjusted hazard ratio (aHR) 2.14; 95% confidence interval (CI) 1.70–2.70 and current and previous TB aHR (95% CI) were 2.70 (1.81–4.04) and 1.51 (1.18–1.93), respectively (40). However, clinical experience to date does not indicate that HIV or TB are major risk factors for COVID-19 death in children, however, analyses of provincial data are ongoing.

With the lockdown and de-escalation of health services that were seen globally, patients have had reduced access to health facilities to collect chronic medications for HIV/TB or other chronic diseases, both due to lockdown regulations and for fear of exposure to COVID-19. Screening services for other common conditions like TB have experienced competition, e.g., gene expert cartridges for TB being used for COVID-19 (69); sputum specimens being prioritized for COVID-19 testing delaying diagnosis of TB and inappropriate use of BCG in response to the unproven theory that BCG may protect against COVID-19 (64). It is anticipated that a major decline in number of TB cases screened and tested has occurred and that disruption in the treatment programs for these diseases will ensue unless the COVID-19 screening and testing program can run simultaneously with HIV/TB programs (70).

The clinical presentation for COVID-19, TB or other respiratory disease, is non-specific. Health care workers in high burden areas must insist on standard screening of TB and HIV in children presenting with respiratory tract illness, in addition to COVID-19 testing. Data is needed to better understand the risk of not only HIV infection but also HIV exposure on COVID-19 risk. In addition, the implications of BCG vaccination, previous TB disease and current co-infection with TB for COVID-19 severity in children requires further investigation.

## Summary

COVID-19 is associated with milder disease in children compared to adults. Despite initial fears over risk for children with chronic lung diseases, current evidence suggests that pre-existing respiratory disease in children does not appear to be a significant risk factor for severe COVID-19. This has important implications for planning with social care and schooling. Longitudinal and larger datasets are still needed to assess risk in children with immunosuppression and interstitial lung diseases, and caution should be taken with these groups. The long-term consequence of COVID-19 on lung health is unknown. In addition, the increasing global recognition of MIS-C as an important cause of COVID-19 related morbidity and mortality in children must not be underestimated, necessitating early recognition and management. Ongoing strict public health measures are essential to reduce the impact of the pandemic on children with respiratory illness and their families. Most importantly, the indirect impacts of the pandemic response on child respiratory health are notable and still likely to be fully realized and quantified. Ensuring children have access to full preventive and care services during this time is priority.

## Author Contributions

DG and AV collated and wrote the final version of the manuscript. HZ, MZ, and M-AD provided additional intellectual input. All authors contributed to researching and writing sub-sections of the article, reviewed, and commented on the final version of the manuscript.

## Conflict of Interest

The authors declare that the research was conducted in the absence of any commercial or financial relationships that could be construed as a potential conflict of interest.
